# Evolution of discrepancies in limb asymmetry in Beckwith–Wiedemann spectrum

**DOI:** 10.1177/18632521251384575

**Published:** 2025-10-15

**Authors:** Ryan D Lopez, Carter E Hall, Jonathan H Sussman, Andrew M George, Charles A Phillips, Carolyn Gerace, Richard S Davidson, Jennifer M Kalish

**Affiliations:** 1 Perelman School of Medicine, University of Pennsylvania, Philadelphia, PA, USA; 2 Division of Orthopaedics, Children’s Hospital of Philadelphia, Philadelphia, PA, USA; 3 Division of Human Genetics, Children’s Hospital of Philadelphia, Philadelphia, PA, USA; 4 Division of Oncology, Children’s Hospital of Philadelphia, Philadelphia, PA, USA; 5 Center for Childhood Cancer Research, Children’s Hospital of Philadelphia, Philadelphia, PA, USA; 6 Department of Biomedical and Health Informatics, Children’s Hospital of Philadelphia, Philadelphia, PA, USA; 7 Departments of Pediatrics and Genetics, Perelman School of Medicine, University of Pennsylvania, Philadelphia, PA, USA

**Keywords:** Beckwith–Wiedemann syndrome, lateralized overgrowth, limb bulk, limb-length discrepancy, limb asymmetry

## Abstract

**Background:**

Beckwith–Wiedemann spectrum is a genetic disorder characterized by lateralized overgrowth, often presenting as limb bulk discrepancy. While limb-length discrepancies are documented in Beckwith–Wiedemann spectrum, the natural history of limb bulk discrepancy and hand/foot asymmetry progression remains less studied. This research examines limb bulk discrepancy progression in children with Beckwith–Wiedemann spectrum and identifies factors influencing its severity.

**Methods:**

A retrospective, single-institution study analyzed 142 children with molecularly confirmed Beckwith–Wiedemann spectrum (imprinting center 2 loss of methylation, imprinting center 1 gain of methylation, and paternal uniparental isodisomy at chromosome 11). Limb measurements (upper arm, forearm, thigh, calf, palm, finger, and foot) were recorded during clinic visits. Linear mixed-effects models assessed relationships between genotype, age, limb bulk discrepancy progression, body mass index, and sex.

**Results:**

The cohort included 90 imprinting center 2 loss of methylation, 41 paternal uniparental isodisomy at chromosome 11, and 11 imprinting center 1 gain of methylation patients. In the imprinting center 2 loss of methylation group, significant limb bulk discrepancy progression occurred in the upper arm (0.47 mm/year), calf (0.53 mm/year), and foot anterior–posterior dimension (0.40 mm/year; all *p* < 0.01). The paternal uniparental isodisomy at chromosome 11 genotype showed greater asymmetry in most regions compared to others (*p* < 0.01), except the middle finger. Asymmetry progression rates were similar across genotypes. Body mass index positively correlated with increased limb bulk discrepancy in the upper arm and calf.

**Conclusions:**

Limb asymmetry in Beckwith–Wiedemann spectrum progresses slowly in specific regions, with genotype and body mass index influencing baseline severity. Patients with paternal uniparental isodisomy at chromosome 11 exhibit greater baseline limb bulk discrepancy, but progression rates are consistent across genotypes, highlighting the need for further research into lateralized overgrowth mechanisms and clinical implications.

**Level of evidence:**

3—Retrospective cohort study.

## Background

**Figure 1.**
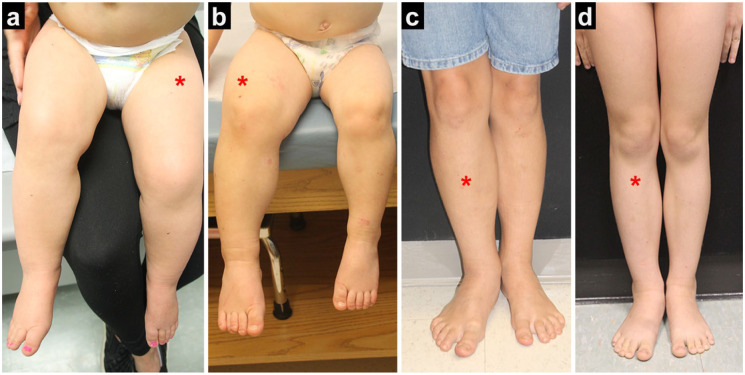
Lateralized overgrowth in the lower extremity (asterisk on larger leg) in patients with IC2 LOM (a), pUPD11 (b, c), and IC1 GOM (d). Images obtained with patient consent. IC1 GOM: imprinting center 1 gain of methylation; IC2 LOM: imprinting center 2 loss of methylation; pUPD11: paternal uniparental isodisomy at chromosome 11.

Beckwith–Wiedemann spectrum (BWS) is an epigenetic disorder characterized by overgrowth in various regions of the body, often presenting with lateralized overgrowth (LO; Figure 1), which refers to the asymmetric overgrowth of one or more areas of the body.^1^ LO manifests in approximately half of patients with BWS, and while most cases of LO are evident at birth, subtle cases may initially go unrecognized.^2,3^ On a molecular basis, BWS results from alterations in imprinting control regions located on chromosome 11p15, with the most frequent alterations including imprinting center 2 loss of methylation (IC2 LOM), imprinting center 1 gain of methylation (IC1 GOM), and paternal uniparental isodisomy at chromosome 11 (pUPD11).^4–6^ Among the molecular subtypes of BWS, LO is most frequent in those with pUPD11.^3,7–10^

The development of LO may have significant downstream orthopedic implications for affected individuals. LO may present clinically as a limblength discrepancy or a limb bulk discrepancy (LBD) between limbs of the upper or lower extremities. Altered gait mechanics can lead to impaired mobility, scoliosis, degenerative disease of the spine, and the development of hip and knee osteoarthritis.^11^ Carli et al. demonstrated that leg length discrepancy tends to progress over time, with the degree of initial leg length discrepancy correlating with more severe future progression.^12^ Consequently, close monitoring is recommended for any detectable discrepancy in patients with syndromic LO, as opposed to the general population, where a leg length discrepancy under 1 cm is often not clinically significant.^12,13^

While leg length discrepancy has been shown to evolve over time in these patients, little has been published on LBD in the BWS, and its natural history remains unclear. LBD can present not only as an esthetic concern, but also with significant quality-of-life implications, making it critical to understand how it evolves to provide accurate counseling and prognostication for patients and their families. This study aims to characterize the evolution of both LBD and hand/foot asymmetry in children with BWS. We aim to identify prognostic factors, if any, that lead to more severe progression of limb asymmetry over time.

## Methods

Following institutional review board approval (protocols IRB 13-010658 and IRB 19-016459), patients with a molecularly confirmed diagnosis of BWS were identified from the database of a single, tertiary-care children’s hospital.^2,4^ Exclusions were performed for patients with a genotype other than the three most common genotypes: IC2 LOM, pUPD11, and IC1 GOM. Genetic testing was primarily performed on blood tissue samples. However, due to the potential for mosaicism, additional tissue types such as skin biopsy from the affected limb, resected tongue tissue, saliva, or normal tissue adjacent to tumors, including Wilms tumor or hepatoblastoma, were analyzed when blood testing was negative.^14^ Patients were also excluded if they had fewer than two clinic visits to be able to draw more meaningful conclusions about the progression of LBD.

The initial criteria query yielded 367 patients. After excluding patients for not having measurement data or duplicate entries, there were 258 unique patients. After excluding patients with fewer than two clinic visits, a total of 142 unique patients were included for final analysis.

**Figure 2.**
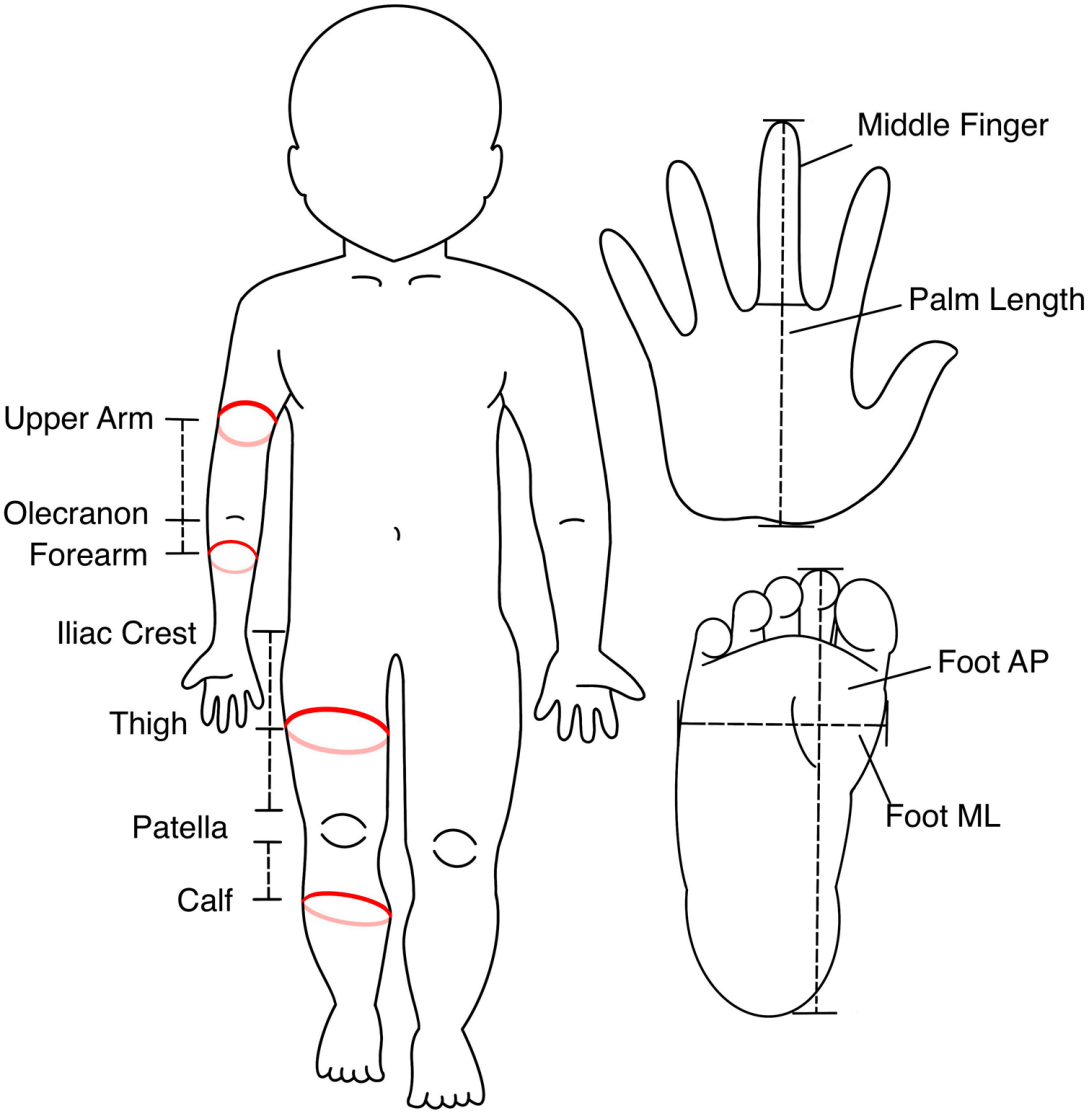
Schematic diagram of bony landmarks, measurement locations, limb circumference, and hand and foot length/width measurements.

Measurements of limb girth were extracted from clinic visit notes in the electronic health record. During every clinic visit with a patient with BWS, circumference measurements are taken at the following locations: upper arm, forearm, thigh, and calf (Figure 2). For leg measurements, the patella was used as the bony landmark from which to measure. The distance in centimeters was measured using a straight-edge ruler to the area of the greatest calf circumference on the left calf. A mark was made with a pen at that distance on the front of the calf. The same distance was measured on the right calf and marked. A tape measure was then placed along the skin around the calf with the top edge touching that mark to measure the bulk of the calf. The tape measure was placed along the skin without constricting the skin to allow for consistency between measurements. The same approach was used to measure the right calf. The approximate midline distance between the top of the patella and the iliac crest was then measured on the left thigh and marked, and the same distance was measured above the right patella and marked. The left thigh and then the right thigh were then measured. When performing measurements, both calves and then both thighs were measured in succession. The forearm distance measurement was taken at the midpoint between the olecranon and the medial distal tip of the radius on the left and marked. The same distance was then measured from the olecranon on the right. The tape measure was then draped around the left and then the right forearm to measure bulk, as was described for the legs. The upper arm distance was measured midway up the upper arm away from the olecranon on the left and marked, and then the same distance from the olecranon on the right. Bulk was measured the same way by draping the tape measure around the arm. Length measurements were also taken of the palm, middle finger, and the foot in both the anterior–posterior (AP) and medial-lateral (ML) dimensions (Figure 2). All measurements were obtained by a consistent clinical team of three genetics providers, including JMK. The team was trained and supervised by JMK throughout the duration of data collection. Training included parallel measurements made by JMK and the two other providers until inter-rater reliability was within 2% for LBD.

The following variables were collected from chart review: age, sex, genotype, height, weight, body mass index (BMI), and ethnicity. The raw difference in bulk circumference or length measurement was calculated and examined in comparison to the age of the patients.

### Statistical analysis

Statistical analysis was conducted in R v4.4.0 (R Foundation for Statistical Computing, Vienna, Austria). To model measurements over time, we applied a linear mixed-effects model using the lmer function of the form: Measurement ~ Age × Genetics + Sex + BMI + (Age | ID). This model’s age, genetic subtype, sex, and BMI as fixed effects, the baseline measurement per patient is a variable effect, and the interaction between age and genetic subtype. The output of the linear mixed-effects model includes the fixed effect (β) and interaction effects. The interaction effects aim to elucidate the difference in the rate of asymmetry progression by genotype. The IC2 LOM cohort was specified as the reference cohort for the model because it is the most common genotype. Outliers were identified by examining the residuals of the initial model. Data points with residuals greater than two standard deviations were excluded. For visualization purposes, both the age and measurement value were adjusted by subtracting their baseline value to demonstrate individual trajectories. Both individual patient trajectories (Figures S1 and S2) and average trends (Figures 1 and 2) were plotted for each genotype group. Chi-square testing was used to determine differences in the demographics of each cohort.

## Results

The demographics of the included patients are listed in Table 1. IC2 LOM was the most common genotype (63%), followed by pUPD11 (29%), then IC1 GOM (7%). There were no significant differences in age at presentation, sex, or race between groups. All cohorts had a majority of female and Caucasian/White patients. The average and median number of clinic visits for each included patient were 3.3 and 3, respectively. The age of included patients spanned from 1 week to 12 years old.

**Table 1.** Demographics.

Demographics	Total (*N* = 142)	%	IC2 LOM (*N* = 90)	%	pUPD11 (*N* = 41)	%	IC1 GOM (*N* = 11)	%	*p*
Median age at first visit (months ± SD)	7.5 ± 21.7		6.3 ± 19.7		8.6 ± 16.0		15.9 ± 43.0		0.163
Sex									0.366
M	53	37.3	36	40.0	15	36.6	2	18.2	
F	89	62.7	54	60.0	26	63.4	9	81.8	
Race/ethnicity									0.360
Caucasian/White	107	75.4	67	74.4	31	75.6	9	81.8	
Black	8	5.6	5	5.6	3	7.3	0	0	
Hispanic	13	9.2	6	6.7	6	14.6	1	9.1	
Asian	2	1.4	1	1.1	0	0.0	1	9.1	
American Native	1	0.7	1	1.1	0	0.0	0	0.0	
Other	7	4.9	7	7.8	0	0.0	0	0.0	
No Response	4	2.8	3	3.3	1	2.4	0	0.0	

IC1 GOM: imprinting center 1 gain of methylation; IC2 LOM: imprinting center 2 loss of methylation; pUPD11: paternal uniparental isodisomy at chromosome 11; SD: standard deviation.

### Limb bulk discrepancy

**Figure 3.**
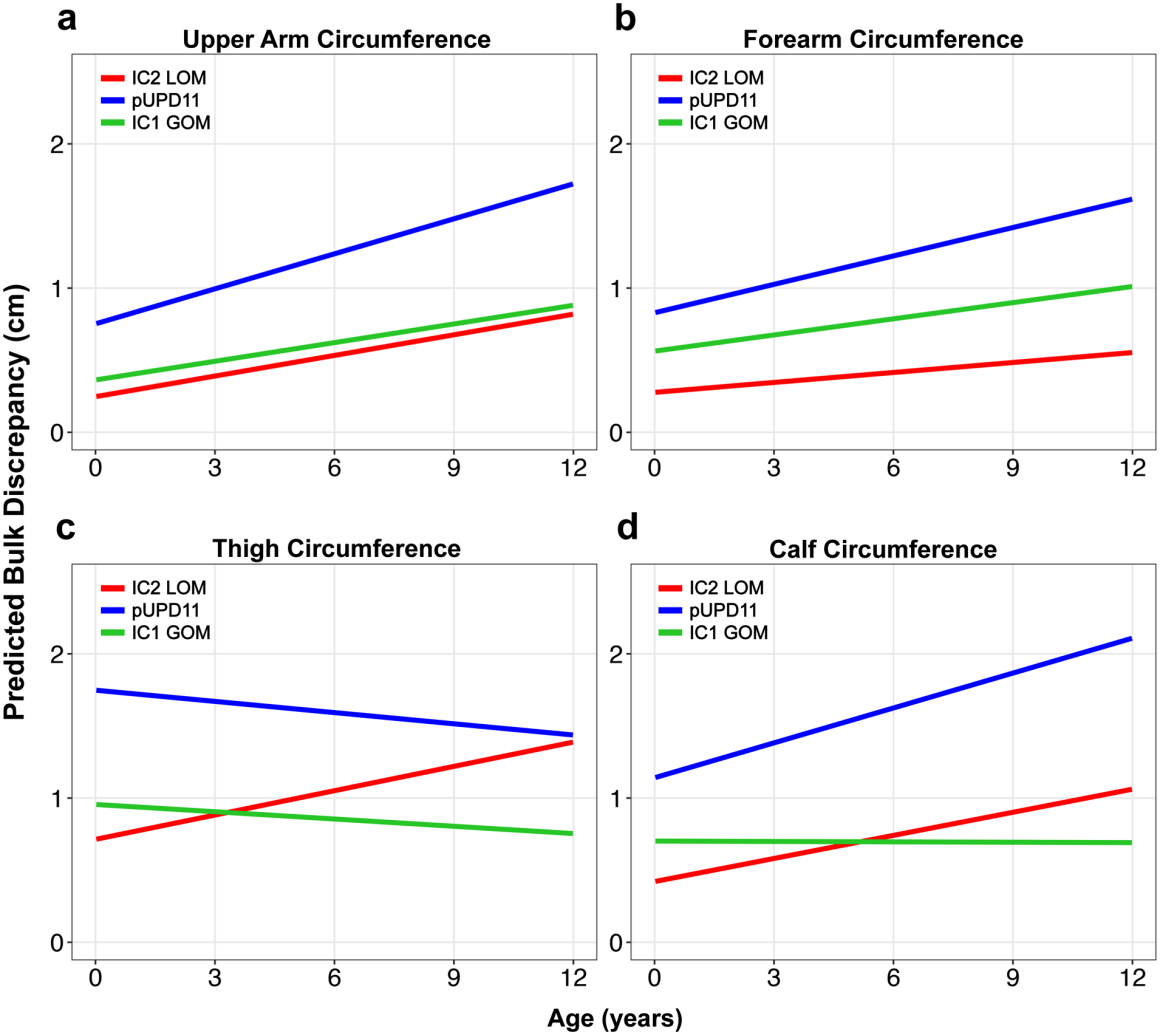
Predicted LBD over time for each genetic subtype based on the linear mixed-effects model in various body parts: (a) upper arm, (b) forearm, (c) thigh, and (d) calf. LBD: limb bulk discrepancy.

**Figure 4.**
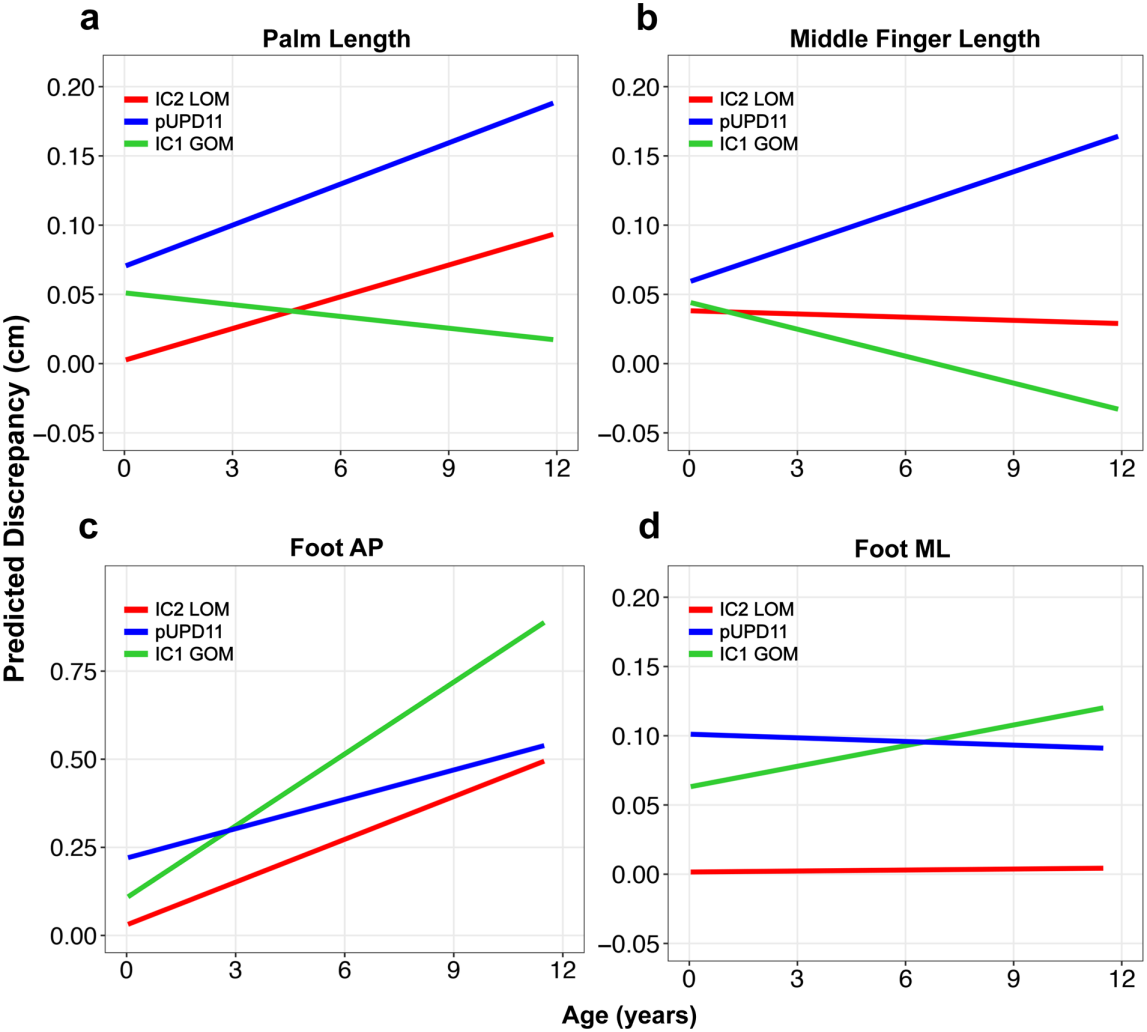
Average LBD change over time for each genetic subtype based on linear mixed-effects model for various locations in the hands/feet: (a) palm, (b) middle finger, (c) foot AP, and (d) foot ML. AP: anterior-posterior; LBD: limb bulk discrepancy; ML: medial-lateral.

In the IC2 LOM cohort, the upper arm circumference difference increased by an average of 0.047 cm/year (*p* = 0.005, Table 2), with notable variability across patients, as shown in Figure S1. Patients with pUPD11 genotype showed a significant fixed effect on upper arm circumference difference (β = 0.51, *p* = 0.005), indicating an average 0.51 cm greater upper arm circumference difference compared to IC2 LOM patients at any given time point. IC1 GOM genotype did not have a significant fixed effect compared to IC2 LOM (β = 0.12, *p* = 0.582). BMI also had a positive association with upper arm circumference difference (β = 0.036, *p* = 0.006). While LBD increased over time in all cohorts, there were no statistical differences in the rate of limb asymmetry progression between any of the genotype cohorts (Figures 3 and 4).

**Table 2.** Linear mixed-effects model results for limb circumference differences.

Factors considered	Upper arm		Forearm		Thigh		Calf	
	Effect	*p*	Effect	*p*	Effect	*p*	Effect	*p*
LBD growth rate^a^ (cm/year)	0.047	**0.005**	0.023	0.162	0.056	0.071	0.053	**0.009**
Fixed effects								
pUPD11	0.505	**0.005**	0.552	**<0.001**	1.034	**<0.001**	0.720	**<0.001**
IC1 GOM	0.116	0.582	0.286	0.153	0.242	0.556	0.281	0.233
Sex (male)	0.097	0.261	0.198	**0.040**	0.066	0.677	0.083	0.455
BMI	0.036	**0.006**	0.024	0.068	0.0.006	0.799	0.037	**0.009**
Interaction terms								
Age: pUPD11	0.033	0.179	0.043	0.076	−0.082	0.081	0.027	0.355
Age: IC1 GOM	−0.005	0.920	0.014	0.762	−0.073	0.344	−0.054	0.300

Significant *p*-values highlighted in bold. BMI: body mass index; IC1 GOM: imprinting center 1 gain of methylation; IC2 LOM: imprinting center 2 loss of methylation; LBD: limb bulk difference; pUPD11: paternal uniparental isodisomy at chromosome 11.

a Growth rate is calculated for the reference cohort, IC2 LOM. This *p* value corresponds to the significance of the relationship between LBD and age.

Forearm circumference difference did not significantly increase over time in the IC2 LOM cohort (β = 0.02, *p* = 0.162). However, patients with the pUPD11 genotype exhibited a larger forearm circumference (β = 0.58, *p* < 0.001). Male sex (β = 0.20, *p* = 0.040) had a significant fixed effect on forearm asymmetry (Table 2). Age and genotype interaction effects did not reach statistical significance (Table 2).

For the thigh circumference difference, the average rate of asymmetry progression was 0.057 cm/year in the IC2 LOM group (*p* = 0.07), though this relationship did not reach statistical significance (Table 3). The pUPD11 genotype had a significant fixed effect (β = 1.02 cm, *p* < 0.001), indicating a mean 1.02 cm greater thigh circumference difference at any given time point compared to the IC2 LOM group. While both pUPD11 and IC1 GOM cohorts exhibited decreases in LBD over time (Figure 3), these differences did not reach the level of significance (pUPD11: β = −0.08 cm/year, *p* = 0.081; IC1 GOM: β = −0.07 cm/year, *p* = 0.344). Sex and BMI fixed effects were also not significant (Table 3).

**Table 3.** Linear mixed-effects model results for hand and foot differences.

Factors considered	Palm		Middle finger		Foot AP		Foot ML	
	Effect	*p*	Effect	*p*	Effect	*p*	Effect	*p*
Discrepancy growth rate^a^ (cm/year)	0.008	0.120	0.001	0.866	0.040	**0.001**	0.001	0.954
Fixed effects								
pUPD11	0.068	**0.009**	0.021	0.323	0.190	**<0.001**	0.100	**<0.001**
IC1 GOM	0.049	0.310	0.006	0.871	0.077	0.460	0.061	0.214
Sex (male)	0.028	0.179	0.005	0.709	0.068	0.084	0.057	**0.015**
BMI	0.005	0.195	0.005	0.089	0.003	0.678	−0.001	0.954
Interaction terms								
Age: pUPD11	0.001	0.763	0.010	0.185	−0.013	0.503	−0.001	0.858
Age: IC1 GOM	−0.010	0.449	−0.006	0.653	0.028	0.417	−0.005	0.677

Significant p-values highlighted in bold. AP: anterior-posterior, BMI: body mass index; IC1 GOM: imprinting center 1 gain of methylation; IC2 LOM: imprinting center 2 loss of methylation; LBD: limb bulk difference; ML: medial-lateral; pUPD11: paternal uniparental isodisomy at chromosome 11.

a Growth rate is calculated for the reference cohort, IC2 LOM. This *p* value corresponds to the significance of the relationship between discrepancy and age.

Calf circumference asymmetry increased at an average of 0.053 cm/year in the IC2 LOM group (*p* = 0.009). Both BMI (β = 0.04, *p* = 0.009) and the pUPD11 genotype (β = 0.72, *p* < 0.001) were significant predictors of greater limb asymmetry (Table 3). While the IC1 GOM cohort tended to decrease over time (Figure 3), the interaction effects of genotype did not statistically affect the progression of LBD.

### Hand and foot asymmetry

Palm and middle finger length differences did not increase with age (Table 3). For the palm length difference, the pUPD11 genotype was the only significant predictor (β = 0.07 cm, *p* = 0.009), while BMI and sex showed no significant effects. For the middle finger length difference, no significant predictors were found. In both the palm and middle finger, the IC1 GOM cohort tended to decrease in discrepancy over time, but these effects did not reach significance (Table 3).

For the foot anterior-posterior (AP) dimension, the asymmetry increased by 0.040 cm/year in the IC2 LOM cohort (*p* = 0.001). The pUPD11 genotype (β = 0.19, *p* < 0.001) was a significant risk factor for greater AP asymmetry, while other variables were not (Table 3). All genotypes tended to increase in foot AP length discrepancy, and there was no difference in the rate of progression over time across genotypes.

In the foot medial-lateral (ML) dimension, there was no relationship between asymmetry and age (0.001 cm/year, *p* = 0.954). The pUPD11 genotype (β = 0.100 cm, *p* < 0.001) and male sex (β = 0.057 cm, *p* = 0.015) were significant predictors for increased fixed difference, while BMI showed no significant association. There was no effect of genotype on the rate of LBD progression in the foot ML dimension (Table 3).

## Discussion

In this study, we investigated the progression of LBD and asymmetry of the hands and feet within a large cohort of patients with molecularly confirmed BWS. Our findings demonstrate that asymmetry tends to increase over time for upper arm and calf circumference and foot AP length. Patients with the pUPD11 genotype tended to have statistically greater asymmetry compared to the most common BWS genotype, IC2 LOM. Despite these findings, the magnitude of asymmetry increase over time was modest, frequently on the order of a fraction of a centimeter per year. Importantly, no significant differences were observed in the rate of LBD progression across genotypes, suggesting that the genetic subtype primarily influences the baseline severity rather than the dynamic progression of LBD over time.

This is the first study to provide insights into LBD progression over time in patients with BWS. For the most common genotype, IC2 LOM, LBD was found to statistically increase over time in the upper arm, calf, and foot AP length. The other measured body parts did not show statistically significant progression. The reason for the differential progression of asymmetry for the upper arm, calf, and foot AP versus other body parts is not presently clear. The level and distribution of somatic mosaicism, which has been implicated as a major determinant of phenotypic severity in BWS, is likely a contributing factor.^15–17^ However, this variable could not be reliably collected in the limb tissues for patients in this cohort. Future studies incorporating tissue-specific mosaicism (where possible) may elucidate the mechanisms driving regional variation in LBD progression. Understanding these patterns is key, as they have implications for both functional outcomes and esthetic concerns for patients and their families.^18^

Genotype-specific differences in BWS phenotypes have been well-documented in prior studies, particularly in relation to tumor risk and leg length discrepancy.^12,16,19^ For example, pUPD11 has been identified as leading to a more severe overgrowth phenotype, including increased tumor risk, and asymmetric limb development when compared to IC2 LOM.^4,12,19–22^ These trends were supported by the findings of this study. The pUPD11 genotype was found to have a significant fixed effect on LBD in all measured body parts except for palm width. This indicates that at any given time point, pUPD11 patients will have a larger LBD compared to the IC2 LOM group; however, this difference does not magnify over time compared to the other genotypes according to our model. These findings diverge from those by Carli et al., who reported genotype-specific differences in leg length discrepancy progression.^12^

Given the more severe presentation of pUPD11 in some cases, close monitoring of LO is recommended, particularly leg length discrepancy, which may lead to potential functional limitations, such as gait abnormalities or asymmetries in walking mechanics.^18^ Leg length discrepancy, which is often treatable through interventions such as orthotics or surgical correction, appears to be a primary contributor to these issues.^18^ The role of LBD in contributing to long-term functional impairment remains unclear due to limited literature on the topic. According to our results, it is unlikely that most patients will develop functional impairments secondary to LBD, considering the modest increases observed in this study. Furthermore, as patients age and their limbs undergo expected growth, the relative magnitude of LBD often decreases in proportion to overall limb size. This trend provides reassurance that while LBD remains a concern, its functional impact may be mitigated over time.

Apart from genotype, this study also investigated the role of sex and BMI on LBD progression. Male sex was found to be a risk factor for increased LBD in the forearm and foot ML width. BMI had a significant fixed effect on LBD in the upper arm, forearm, and calf. This suggests that increased muscle and fat deposition may tend to exacerbate observed asymmetries. Conversely, in our group’s clinical experience, some patients indicate that weight loss and an increased muscle-to-fat ratio may enhance perceived LBD asymmetry. For patients with more severe LBD, it is important to identify modifiable factors that may alleviate their asymmetry. This is important to potentially mitigate not only esthetic concerns but also altered functional mechanics, which may have downstream orthopedic consequences.^18^ However, it is not presently known whether there is a critical threshold LBD that alters walking mechanics in a way that would predispose patients to orthopedic disease as they age.

While not directly investigated in our study, the underlying tissue composition contributing to increased limb girth in patients with BWS remains an important question. However, a 2025 study by Gazzin et al. employed total body dual-energy X-ray absorptiometry to objectively quantify tissue composition in patients with LO.^23^ In individuals with BWS, the affected regions demonstrated significantly greater proportions of both muscle and bone tissue compared to the contralateral, unaffected side, indicating a pattern of musculoskeletal overgrowth rather than expansion of adipose fat.^23^

Our use of linear mixed-effects modeling provided critical insights into the progression of LBD in patients with BWS, allowing us to account for longitudinal changes within individuals and variability between genetic subtypes. By incorporating covariates such as BMI, sex, and repeated measures, the model controlled for key confounders, enabling a clearer understanding of genotype-specific trends. These findings reinforce the importance of stratifying patients by genotype in clinical monitoring, as they provide a foundation for tailoring counseling regarding LBD progression in different risk-stratified groups. Clinically, this information equips providers with objective data to offer families clearer expectations about how asymmetry may evolve over time. This can reduce uncertainty, enhance anticipatory guidance, and support more informed decision-making during the patient’s subsequent care.

### Limitations

Although this is the largest cohort of patients with BWS with LBD measurements to date, there are several limitations to acknowledge. First, there was a notable decrease in clinical follow-up for patients entering adolescence. Thus, it is not possible to draw conclusions on LBD progression into skeletal maturity from this study. Further studies should investigate how LBD in this cohort is affected by the growth acceleration observed during puberty. This study is also reliant on our model, producing a population-level estimation of LBD progression. However, as shown in Figures S1 and S2, there is significant individual-level variation, and certain patients may have large exacerbations of LBD. Factors leading to extreme progression of LBD have yet to be clarified and may require data with adolescent representation to capture all periods of pronounced growth. In addition, the relationship between BWS phenotypic severity and blood mosaicism has been demonstrated, which may have insights for this line of study.^4,24^ Finally, while all LBD measurements were performed by three genetics providers, JMK plus two providers trained by JMK, we acknowledge this as a potential source of variability, but all followed the standardized measurement protocol, as detailed in the methods section.

## Conclusions

In conclusion, asymmetry increases slowly over time in the upper arm, calf, and foot anterior–posterior (AP) in patients with BWS. Notably, the pUPD11 genotype is associated with increased asymmetry in patients with BWS compared to other common genotypes; however, the rate of asymmetry progression over time is consistent across genotypes. This study highlights the critical role of genetic stratification in understanding and managing asymmetry progression in BWS. By characterizing the trajectory of limb asymmetry, our findings provide a framework for informing the clinical prognosis and tailoring monitoring strategies to individual patients. These results not only contribute to the growing body of literature on genotype-phenotype correlations in BWS but also pave the way for more personalized approaches to care.

## Supplemental Material

sj-docx-2-cho-10.1177_18632521251384575 – Supplemental material for Evolution of discrepancies in limb asymmetry in Beckwith–Wiedemann spectrum Supplemental material, sj-docx-2-cho-10.1177_18632521251384575 for Evolution of discrepancies in limb asymmetry in Beckwith–Wiedemann spectrum by Ryan D Lopez, Carter E Hall, Jonathan H Sussman, Andrew M George, Charles A Phillips, Carolyn Gerace, Richard S Davidson and Jennifer M Kalish in Journal of Children's Orthopaedics

sj-pdf-1-cho-10.1177_18632521251384575 – Supplemental material for Evolution of discrepancies in limb asymmetry in Beckwith–Wiedemann spectrum Supplemental material, sj-pdf-1-cho-10.1177_18632521251384575 for Evolution of discrepancies in limb asymmetry in Beckwith–Wiedemann spectrum by Ryan D Lopez, Carter E Hall, Jonathan H Sussman, Andrew M George, Charles A Phillips, Carolyn Gerace, Richard S Davidson and Jennifer M Kalish in Journal of Children's Orthopaedics
