# Supplementary material for: Evolution of discrepancies in limb asymmetry in Beckwith–Wiedemann spectrum
Source: J Child Orthop. 2025 Oct 15;19(6):463–72. doi: 10.1177/18632521251384575 (PMC12528058; doi:10.1177/18632521251384575)
Supplement: sj-docx-2-cho-10.1177_18632521251384575 – Supplemental material for Evolution of discrepancies in limb asymmetry in Beckwith–Wiedemann spectrum [file sj-docx-2-cho-10.1177_18632521251384575.docx]

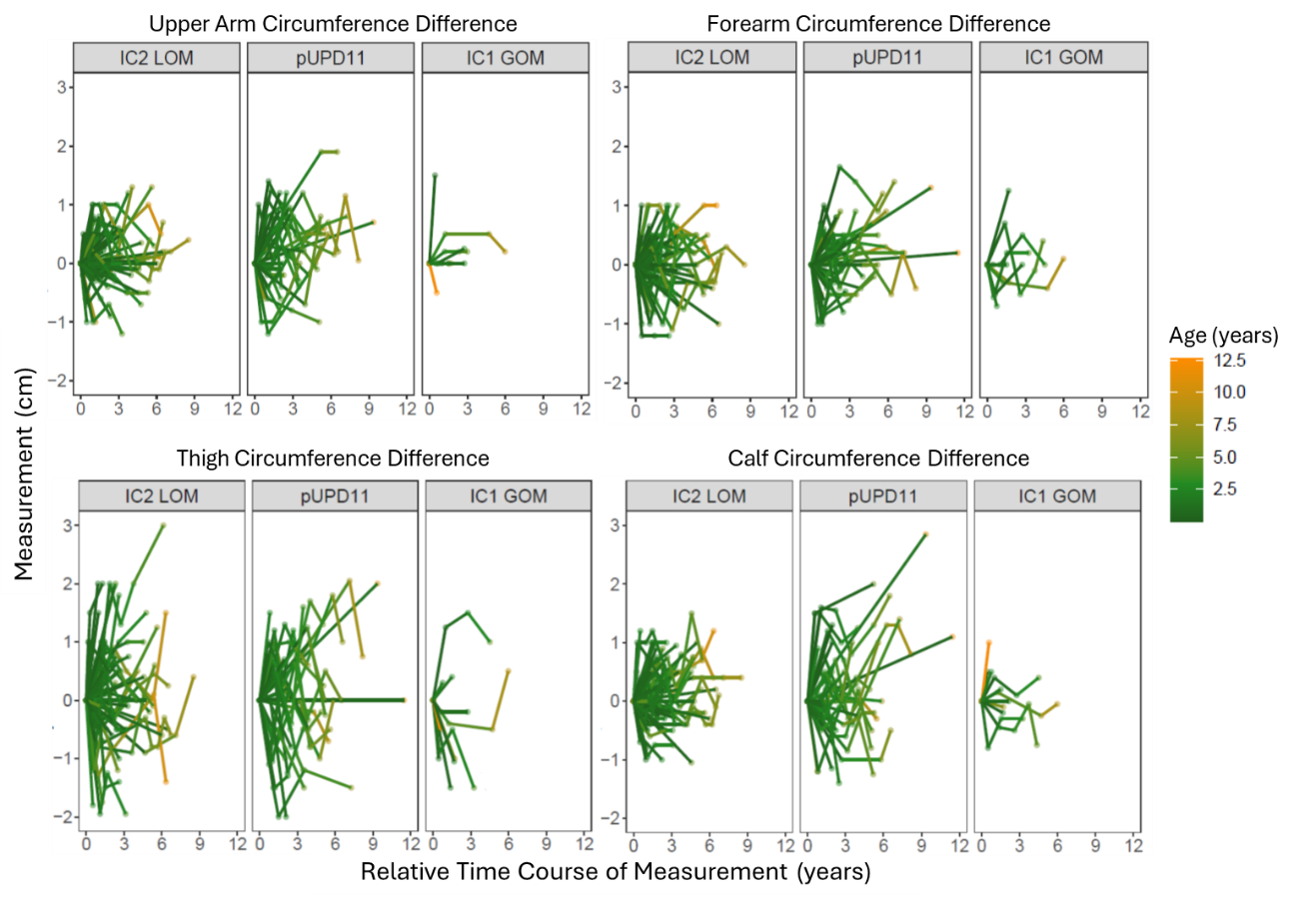


Figure S1. Individual patient trajectories of limb bulk differences (LBD) over time by genetic subtype in various locations: A) upper arm circumference, B) forearm circumference, C) thigh circumference, and D) calf circumference. Different lines indicate measurement trajectories in different individual patients. Relative time course of measurement accounts for the length of time over which the patient was returning to clinic for measurements. Actual age is indicated by the color gradient.


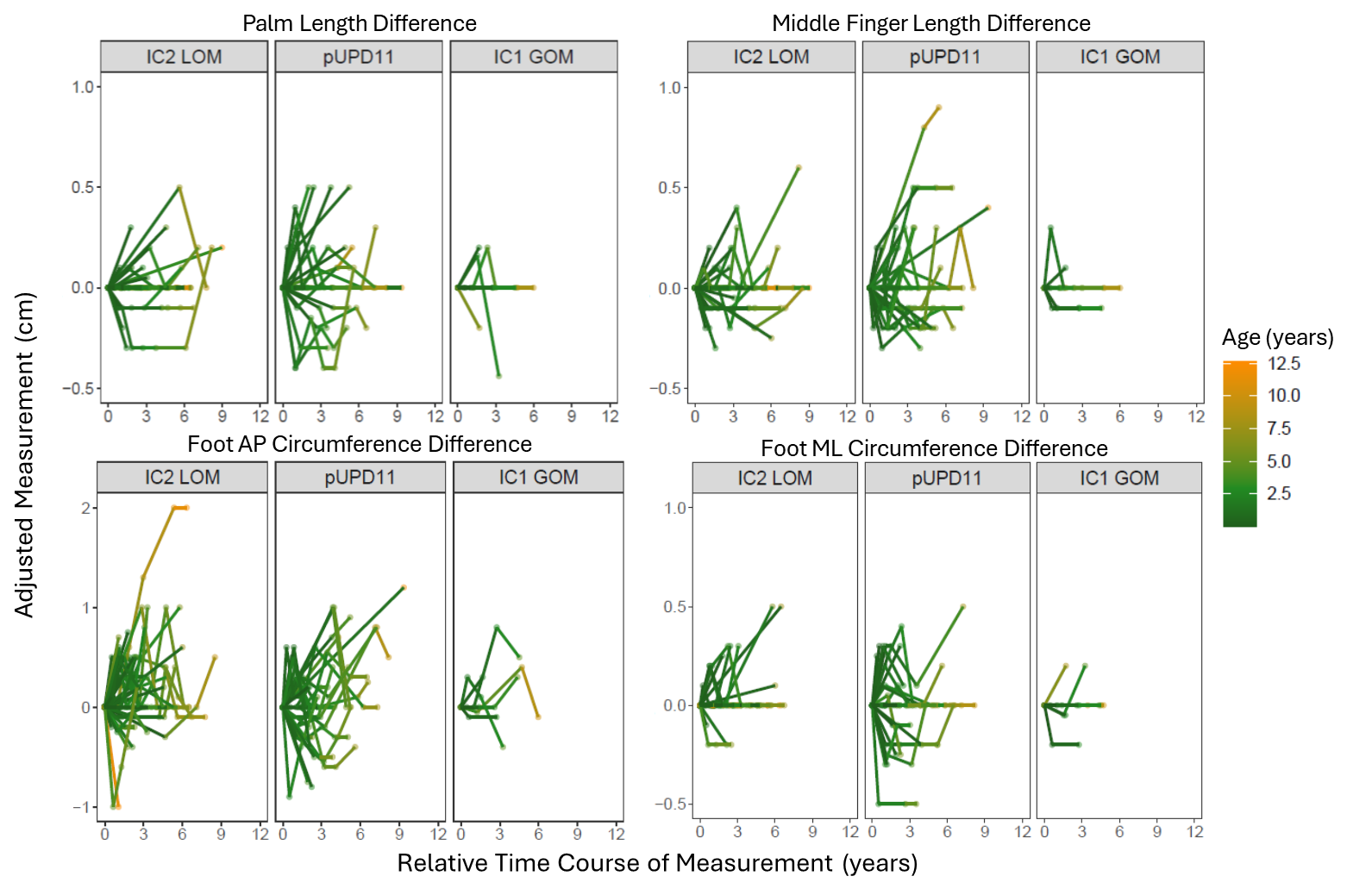


Figure S2. Individual patient trajectories of hand and foot measurement asymmetry: A) palm length circumference, B) middle finger length, C) foot anteroposterior (AP) length, and D) foot mediolateral (ML) length. Different lines indicate measurement trajectories in different individual patients. Relative time course of measurement accounts for the length of time over which the patient was returning to clinic for measurements. Actual age is indicated by the color gradient.
